# Comparative characterization of two monoclonal antibodies targeting canine PD-1

**DOI:** 10.3389/fimmu.2024.1382576

**Published:** 2024-05-08

**Authors:** Mikolaj Kocikowski, Katarzyna Dziubek, Katarzyna Węgrzyn, Vaclav Hrabal, Filip Zavadil-Kokas, Borivoj Vojtesek, Javier Antonio Alfaro, Ted Hupp, Maciej Parys

**Affiliations:** ^1^ International Centre for Cancer Vaccine Science, University of Gdansk, Gdansk, Poland; ^2^ The Royal (Dick) School of Veterinary Studies and The Roslin Institute, University of Edinburgh, Midlothian, United Kingdom; ^3^ Intercollegiate Faculty of Biotechnology of University of Gdansk and Medical University of Gdansk, Gdansk, Poland; ^4^ Research Centre for Applied Molecular Oncology, Masaryk Memorial Cancer Institute, Brno, Czechia; ^5^ Department of Biochemistry and Microbiology, University of Victoria, Victoria, BC, Canada; ^6^ Institute for Adaptive and Neural Computation, School of Informatics, University of Edinburgh, Edinburgh, United Kingdom; ^7^ Institute of Genetic and Molecular Medicine, University of Edinburgh, Edinburgh, United Kingdom

**Keywords:** cancer immunotherapy, immune checkpoint, monoclonal antibody, canine cancer, comparative oncology, veterinary oncology, PD-1, PD-L1

## Abstract

Monoclonal antibodies targeting immune checkpoints have revolutionized oncology. Yet, the effectiveness of these treatments varies significantly among patients, and they are associated with unexpected adverse events, including hyperprogression. The murine research model used in drug development fails to recapitulate both the functional human immune system and the population heterogeneity. Hence, a novel model is urgently needed to study the consequences of immune checkpoint blockade. Dogs appear to be uniquely suited for this role. Approximately 1 in 4 companion dogs dies from cancer, yet no antibodies are commercially available for use in veterinary oncology. Here we characterize two novel antibodies that bind canine PD-1 with sub-nanomolar affinity as measured by SPR. Both antibodies block the clinically crucial PD-1/PD-L1 interaction in a competitive ELISA assay. Additionally, the antibodies were tested with a broad range of assays including Western Blot, ELISA, flow cytometry, immunofluorescence and immunohistochemistry. The antibodies appear to bind two distinct epitopes as predicted by molecular modeling and peptide phage display. Our study provides new tools for canine oncology research and a potential veterinary therapeutic.

## 1 Introduction

The tumor-protective ability of cancer cells to modulate the host’s immune reactions through immune checkpoint (IC) receptors has been targeted with immune checkpoint inhibitors (ICI). These therapeutic antibodies most commonly prevent interaction between Programmed cell death protein 1 (PD-1) inhibitory receptor of the immune cells and its most studied ligand, Programmed cell death ligand 1 (PD-L1) (1, 2). The ligand, when expressed by healthy cells, is instrumental for maintaining self-tolerance. However, PD-L1 expression by multiple human cancer types is well-documented and sufficient to induce an immunosuppressive tumor microenvironment (3). The ICI monoclonal antibodies (mAbs) Nivolumab and Pembrolizumab significantly increase survival of cancer patients in multiple cancer types. Treatment response is especially marked in cases with high tumor PD-L1 expression (4). However, a large subset of patients must be excluded from treatment, do not benefit from it, or experience disease hyperprogression (5). Additionally, delayed adverse effects of autoimmune character are common (6, 7).

The failure to anticipate and mitigate such issues in the drug development process demonstrates a dire need for a new, more adequate preclinical research model (8). We as well as others have described the shortcomings of laboratory mice for immunological studies (5, 9). While a fully developed immune system is necessary to recapitulate human treatment response, laboratory mice live in artificial habitats, lacking natural antigenic stimulation. The composition of the microbiome is known to influence immunotherapy outcomes (10), yet laboratory mice do not possess a natural one. Their artificially induced tumors mimic neither the real cancer heterogeneity nor the complex mutational history or the mutual cancer-host immunoediting, all characteristics of human cancer.

By contrast, canine cancers, spontaneous, heterogeneous, developing along with a fully functional immune system, are highly similar to human equivalents and constitute a model uniquely suited for immunotherapy evaluation (5, 11, 12). They are not rare either, with approximately 25% of all dogs dying of cancer (13). Dogs resemble humans in terms of their body size, and their tumors present similar immune infiltration (14). In certain types of cancer, such as osteosarcoma, the canine patient pool outnumbers the human patient population by a ratio of 10 to 1. This significant difference presents an opportunity for statistically robust studies of such malignancies (14). What’s important for antibody-based therapy, the canine antigen receptor locus is more similar to the human one than the murine one is (15). Traditionally, phylogenetic analyses have classified humans (Primates) and mice (Rodentia) within the same clade, Euarchontoglires, while placing dogs (Carnivora) in a separate clade, Laurasiatheria. However, more recent molecular studies challenged this view, proposing that humans and dogs share a closer evolutionary relationship (15, 16). Therapeutic trials involving dogs - our genetic ‘cousins’ - could facilitate thorough immunotherapy evaluation, leading to higher drug success rates, safer human therapeutics and cost-effective development of lacking veterinary treatments.

The PD-1 receptor is expressed by canine immune cells (17, 18) and the PD-L1 expression has been observed in various cancers of companion animals including dogs (19–21). To date, a few groups developed monoclonal antibodies targeting canine PD-1 or PD-L1. Choi et al. developed an anti-canine PD-L1 antibody that blocked the PD-1 checkpoint *in vitro* (22). Maekawa et al. identified rat anti-bovine PD-L1 antibodies that recognized canine PD-L1 and blocked PD-1/PD-L1 interaction (19). The antibody was recombined to create a canine-rat chimera and subsequently tested in seven dogs with oral malignant melanoma (OMM) and two with undifferentiated sarcoma. In this study, exploratory in nature, responses were observed in two of these dogs (23). Most recently, Oh and colleagues developed a blocking anti-canine PD-L1 antibody, which was comprehensively tested through *in vitro, ex vivo* and *in vivo* assays, describing its therapeutic potential, pharmacokinetics and safety profile in dogs (24). Regarding the PD-1, Coy et al. developed a panel of 11 murine antibodies against this canine receptor and characterized 2 of them (17). Nemoto and colleagues developed rat antibodies targeting canine PD-1 and PD-L1; the PD-1 mAb was subsequently caninized and tested in a clinical trial with negative results. This commercial collaboration has not yet resulted in new veterinary drugs (25). As of today, the niche for successful veterinary immune checkpoint blockers remains empty.

Here, we present the characterization of two novel monoclonal antibodies targeting canine PD-1 (cPD-1) - PD1-1.1 (26) and PD1-2.1. The antibodies bound cPD-1 specifically and with strong affinity demonstrated by sub-nanomolar binding in SPR. Both inhibited the PD-1/PD-L1 interaction in a competitive ELISA, with different apparent mechanisms. Furthermore, we scrutinized their capability to detect cPD-1 in Western Blot, ELISA, Flow Cytometry, Immunohistochemistry (IHC) and immunofluorescencent cell staining. Additionally, we conducted computational epitope modeling and phage-based isotope mapping. In both methods the two antibodies appeared to bind distinct chemical sites. The qualities of the PD1-1.1 and PD1-2.1 antibodies position them as promising therapeutic options and complementary tools for advancing comparative oncology research.

## 2 Materials and methods

### 2.1 Generation of anti-canine PD-1 monoclonal antibodies

Canonical PD-1 protein sequences were obtained from Ensembl for human, dog, mouse, Norwegian rat, and rabbit. The canine sequence was compared to others with Protein Blast. The highest similarity score was found between canine and human protein variants, followed by rabbit, rat and mouse. The mouse sequence was least similar, rendering the mouse the best model for raising antibodies against human PD-1. Development of high-affinity antibodies against the target protein would be less probable in a species that produces a nearly identical protein naturally (27).

The antibody generation and production were carried out by Moravian Biotechnology Ltd. (Brno, Czech Republic) under the animal license number 14828/2010–17210 falling under European Union law. The process was performed as previously described (26). Briefly, Balb/c mice were immunized with a recombinant protein fusing the complete canine PD-1 sequence and a his-tag (Sino Biological). Upon reaching high antibody titer in serum the mice were sacrificed, splenocytes were collected, and subsequently fused with SP2 mouse myeloma cells. Conditioned media from the culture of individual hybridoma clones were screened for recognition of recombinant canine PD-1 with a human Fc-tag (Sino Biological). Two best binders, coming from clones cAb1910 and cAb1911, were chosen for further study and named PD1-1.1 and PD1-2.1, respectively. For the experimental assays, the antibodies were purified by Fast protein liquid chromatography (FPLC; Cytiva ACTA) on protein A columns (GE Healthcare), eluted with high salt and cleared on desalting columns (Zeba Spin). Antibody aliquots were stored in PBS at a concentration approximating 1mg/L, with or without (for cell-based assays) 0.1% sodium azide as a preservative. All aliquots were stored frozen at -20°C.

### 2.2 Isotyping

The antibodies were isotyped with a Pierce Rapid Antibody Isotyping Kit (Thermo Scientific, #26179) according to the manufacturer’s protocol.

### 2.3 Cell culture

U-2 OS (U2OS) human osteosarcoma cell line was purchased from Elabscience (#CL-0236). The U2OS line was chosen for its reliable growth in our laboratory. U-2 OS cells were cultured in modified McCoy’s 5A medium (Gibco, #16600082), and HEK293 cells in DMEM medium (Gibco, #10567014). Both media were supplemented with 10% FBS (Gibco, #10500064) and 100UI/ml Penicillin-Streptomycin (Gibco, #15140122). Cells were grown in a humidified atmosphere supplemented with 5% CO2 at 37°C.

### 2.4 Stable PD-1+/- cell lines

To create a stable expression of recombinant protein 2*10^5 cells were seeded per well of a 6-well plate 24 hours before transfection. The transfection was performed using 6.75µl of Attractene transfection reagent (Qiagen, #301005) mixed with 1.8µg of pcDNA 3.1 plasmid vector encoding either an empty vector or canine PD-1 (Thermo Scientific). To create stable cell lines, cell culture media were replaced with selection media containing antibiotics 24 hours after the transfection. Control cells transfected with an empty vector were treated with 100 µg/ml Hygromycin B (Gibco™, #10687010), while PD1-overexpressing cells were selected using 400 µg/ml Geneticin (Roche, #G418-RO) for 2 weeks.

### 2.5 Protein isolation and Western Blotting

U2OS cells were washed two times with PBS and lysed with CelLytic™ M (Sigma-Aldrich, #C2978) mixed with the manufacturer-recommended amount of protease inhibitors (Sigma-Aldrich, #P8340-1ML). Lysates were incubated on ice for 20 minutes and centrifuged for 15 minutes at 14,000 x g at 4°C. Next, samples were denatured by boiling for 5 minutes at 95°C in reducing conditions. Subsequently, 30µg of total protein was separated by SDS-PAGE, where samples were run along a PageRuler Plus Prestained 10-180kDa protein ladder (Thermo Scientific, #26616). The separated proteins were transferred to a nitrocellulose blotting membrane (Amersham Protran) using a wet blotting system (Bio-Rad). The membranes were blocked for 1 hour at RT in 5% skimmed milk diluted in 0.1% Tween-20 in Tris-buffered saline (TBST). Subsequently, membranes were incubated overnight with 1:1000 dilution of either PD1-1.1 or PD1-2.1 antibody, washed three times with TBST and incubated for 1 hour at RT with 1:5000 dilution of HRP-conjugated anti-mouse secondary antibody (abcam, #ab6728). To reprobe the membranes for ß-actin, Restore Plus Western Blot Stripping Buffer (Thermo Scientific, #46430) was used. Following three more washes with TBST, membranes were re-probed with an anti-ß-actin antibody (Abcam #6276, dilution 1:10000), visualized using ECL substrate (Westar Antares, CYANAGEN) and the same lanes were imaged once again with ChemiDoc imaging system (Bio-Rad).

### 2.6 Species specificity by Western-Blot

To assess the cross-reactivity of the characterized antibodies with human PD-1, HEK293 cells, which naturally do not exhibit considerable PD-1 expression, were transfected with either a human PD-1 expression vector containing V5 and Twin-Strep tags (V5-TS-PD1) or the canine PD-1 expression vector. Transfection, cell harvesting, and Western Blotting were performed as previously described. The PD1-1.1 and PD1-2.1 antibodies were tested against the lysates containing PD-1 from both species. Additionally, an antibody against human PD-1 was used as a positive control for the presence of hPD-1 (eBioscience, #14-2798-82). All antibodies were used at a dilution of 1:1000. The membranes were reprobed for ß-actin using the same procedure as described earlier.

### 2.7 PD-1 binding ELISA

A 96-well assay plate (ThermoFisher/Nunc, #442404) was coated overnight at 4°C with 9.4µg of rcPD-1 protein (approximately 98ng per well; Sino Biological, #70109-D08H) diluted in PBS (0.25mg/mL). The plate was washed with PBST (PBS with Tween-20 at 0.1%). All washes were performed three times with 400µl PBST/well. Next, the plate surface was blocked with a blocking buffer (BB) consisting of 3% BSA (Sigma-Aldrich, #A3059) in PBST for 1h. All incubations were performed at room temperature. Serial dilutions of the tested antibodies were prepared in BB and transferred to the assay plate at 100µl/well for a 1-hour incubation. Upon a wash, an HRP-conjugated rabbit polyclonal anti-mouse detection antibody (Agilent/Dako, #P0260; discontinued) diluted in BB was added at 100µl per well. After the final wash, 150µl of TMB (Merck, #ES022) was added, the plate was incubated in darkness at RT with shaking for 30min. Subsequently, absorbance was measured at 650nm using a Varioskan LUX multimode microplate reader (ThermoFisher). Data was pre-processed in Microsoft Excel to subtract blank values and compute the means and standard deviation. The EC50 values were computed and final results visualized using GraphPad Prism.

### 2.8 Flow cytometry

Cells were trypsinized, washed two times with PBS and stained for 30 minutes protected from light at RT with 1:500 dilution of either PD1-1.1 labeled with PE (Abcam, #ab102918) or PD1-2.1 conjugated with APC (Abcam, #ab201807). The antibodies were conjugated with fluorophores earlier as described in the manufacturer’s protocols. Staining with isotype control antibody (BioLegend, #400263) conjugated with PE or APC fluorophore was used as a negative control. Subsequently, cells were washed two times with PBS and analyzed with BD FACSAria II cell sorter (BD Biosciences). Results were analyzed with FlowJo v10.8.1 flow cytometry analysis software (BD Biosciences) with implementation of doublet discrimination.

### 2.9 Surface plasmon resonance

The affinity of the PD1-1.1 and PD1-2.1 to cPD-1 was assessed by surface plasmon resonance (SPR) using a Biacore T200 (GE Healthcare) instrument as described in the manufacturer’s manual. A HEK-produced recombinant canine PD-1 (rcPD-1; 17.7 kDa) protein, residue Met1-Leu169, with a C-terminal His-tag was purchased from Sino Biological (#70109-D08H). Protein purity was > 85% as determined by SDS-PAGE, and the protein was formulated by lyophilization from sterile PBS, pH 7.4. rcPD-1 was reconstituted in sterile water (at a concentration of 0.25mg/mL) and diluted in HBS-EP (150 mM NaCl, 10 mM HEPES pH 7.4, 3 mM EDTA, 0.05% Surfactant P20). The same buffer was used as a running buffer for further analysis. cPD-1 was immobilized on CM5 Series S Sensor Chips (Cytiva) using amine-coupling chemistry at a density of 330RU on flow cell 2 and flow cell 1 was left blank to serve as a reference surface. Ultra-LEAF™ Purified Mouse IgG2a antibody was used as an isotype control (Biolegend, #400263). Both PD1-1.1 and PD1-2.1 solutions but not the isotype control contained 0.02% sodium azide. Tycho instrument (Nanotemper) was used to confirm the stability of tested antibodies in the given buffer and temperature. Analyte weights were predicted based on the amino acid sequence: PD1-1.1 - 146.33kDa, PD1-2.1 - 145.93kDa, isotype control - estimated as 146kDa considering the same species and isotype (28). An approximate molecular weight of 146kDa was used for all calculations. To collect kinetic binding data, the analytes in the running buffer were injected into the flow cells at concentrations of 0, 2.5, 5, 10, 50, 100, 200, and 400 nM. The flow rate was 30 µl/min, injection volume was 120 µl and the temperature was stabilized at 25°C. The sensor chip surface was regenerated with 10 mM glycine pH=1.5. Results were analyzed using Biacore T200 Evaluation Software 3.1 (GE Healthcare). The data were fit to a 1:1 binding model. The results are presented as sensorgrams obtained after subtracting the signal from the reference cell and the signal after buffer injection.

### 2.10 Blocking ELISA

To test the capacity of the PD1.1 and PD2.1 antibodies to block the therapeutically important binding between canine PD-1 receptor and PD-L1 ligand, an ELISA assay was developed and optimized. The recombinant canine PD-1 protein (rcPD-1) with a C-terminal polyhistidine tag (Sino Biological, #70109-D08H) and recombinant canine PD-L1 (rcPD-L1) extracellular domain (ECD) with a C-terminal Fc-tag (Sino biological, #70110-D02H) lyophilized from PBS with protectants were reconstituted in sterile water according to the manufacturer’s instruction, reaching 0.25mg/mL protein concentration. Aliquots were prepared containing 6.25µg of protein each, and were stored at -20°C. Both proteins were originally produced in HEK293 cells. Eukaryotic expression system is crucial for the post-translational modifications (PTMs) of these proteins, such as glycosylation, which affect the protein characteristics and binding. While some researchers use variants of these proteins produced in bacterial expression systems for their low cost, we do not consider data on the PD-1/PD-L1 interaction and its blocking reliable, if obtained using bacterial protein products. F96 Maxisorp Nunc Immuno plate (Thermo Scientific, #442404) was coated overnight at 4°C with 100µl of coating solution per well, containing 125ng of rcPD-1 per well (1.25µg/mL) in PBS (in-house) as a coating buffer. The plate was washed 3x with 400µl PBST (PBS + Tween-20 at 0.01%) and blocked with a blocking buffer (BB). BB consisted of 3% Bovine Serum Albumin (BSA, protease free, essentially globulin free, Sigma, #A3059-100G) in PBST. Upon 90min of blocking, the samples were loaded at 100µl. The perimeter rows of the plate were not used, and were filled with an equal volume of PBS on this and further stages to avoid temperature gradient affecting all the processes. Ultrasensitive TMB substrate (Merck, #ES022-500ML) was brought to room temperature. Samples were prepared by serial dilutions in BB, starting from azide-preserved antibody stocks in PBS at original concentrations of 1mg/mL for PD1.1 and 1.1mg/mL for PD2.1. Controls were loaded with BB. Upon 60min of incubation at room temperature (RT) with the plate covered, another wash followed (all washes were done in the same way), the plate was dried upside down on a paper towel, and 100µl of rcPD-L1 dilution was added to all wells, except the “background signal” control and the perimeter rows. PD-L1 was diluted in BB at the concentration of 7.81 µg/mL (781ng/well). After 1h incubation at RT and a wash, a detection antibody was applied at 100µl/well, 1:500 concentration. This secondary antibody was a mouse anti-human-IgG1 antibody conjugated to horseradish peroxidase (HRP; Invitrogen, #A10648), meant to detect the Fc-tag and hence the PD-L1 that remained bound to the PD-1, whenever the PD-1 binding site was not blocked by the tested antibodies. After a 1h incubation and a wash, TMB substrate was added at 100µl to all wells, and the plate was put on a plate shaker for 50min. Next, 100µl of a STOP solution (0.16M sulfuric acid) was added to all wells, and the yellow reaction product was measured by absorbance of the 450nm wavelength using Varioskan Lux (Thermo Scientific) reader and Skanit RE 6.0.1 software. Results were calculated in Microsoft Excel. The curve plot and IC50 values were obtained with an AAT Bioquest IC50 Calculator (aatbio.com/tools/ic50-calculator), after subtracting the background signal.

### 2.11 Immunohistochemistry

Immunohistochemistry (IHC) was performed on FFPE tissue sections using the Leica Bond Research Detection Kit (Leica Biosystems), according to the standard manufacturer’s protocol. Staining was performed on a BOND-RX Multiplex IHC autostainer (Leica Biosystems), with the following settings: sample preparation - ‘bake and dewax’, staining - ‘routine EnVision mouse’, HIER protocol - ‘HIER 20 min with ER1’. The PD-1 antibody was used at a 1:100 dilution (10µg/mL).

### 2.12 Confocal microscopy of immunocytochemistry

800,000 cells were seeded on 18mm coverslips in a 6-well plate and incubated for 24 hours. Subsequently, cells were fixed with 4% paraformaldehyde for 15 minutes, washed three times with PBS and permeabilized with 0.1% Triton X-100 for 10 minutes. Slides were blocked with 10% goat serum for 1 hour and stained for 1 hour with 100µl of either PD1-1.1 or PD1-2.1 antibodies diluted 1:100 in 0.1% goat serum. Slides were washed five times in 0.1% PBST, 2 minutes each time and incubated for 1 hour protected from light with 100µl of 1:500 dilution of Alexa FluorTM 488 secondary antibody (Invitrogen, #A32723). Subsequently, samples were washed five times with PBST, mounted with ProLong™ Diamond Antifade Mountant with DAPI (Invitrogen, #P36966) and left overnight to dry protected from light. Imaging was performed with Olympus Fluoview FV3000 confocal laser scanning microscope using 63x oil immersion lens. All images were acquired using the same settings for laser power, voltage and gain.

### 2.13 PD-1 epitope modeling for PD1-1.1 and PD1-2.1

The variable domains of both antibodies were modeled with the antibody-specific AbodyBuilder2 tool (29). The canine PD-1 sequence was obtained from Ensembl and was identical to the UniProt sequence (A0A8I3PL99). It is important to note that the Ensembl and UniProt annotations of the canine PD-1 gene were modified when this study was undergoing. The (E2RPS2_CANLF) variant used in some earlier analyses was recalled; additionally, another, longer and differing canine PD-1 sequence available at UniProt ID (A0A8I3PR61) which might be preferable for further studies. However, the three sequences do not differ in the extracellular Ig-like region that was analyzed and modeled in this study. The sequence was divided into domains through ClustalO alignment (30) to the human PD-1 sequence, richly annotated on UniProt (Q15116). Additionally, the canine PD-1 domains were validated with InterPro (31). The identified extracellular, Ig-like receptor domain was submitted for modeling with Phyre2 (32). Each antibody domain was docked to the PD-1 receptor domain using ClusPro2.0 in the antibody mode with masking of the non-CDR regions (33, 34). For each antibody domain, binding models were ranked by the corresponding cluster sizes, and the top most-probable binding model was chosen. Such obtained model was re-exported to a PDB file in PyMol (35), to contain a single-object with three chain labels (L,H, and A for light chain, heavy chain and PD-1). The PDB file was analyzed with PISA (36) to identify the key interface residues. Results were visualized in PyMol.

### 2.14 Phage display-based epitope mapping and data analysis

Epitope mapping using phage display involved one round of panning. Ph.D.-12 Phage Display Peptide Library (New England Biolabs, MA, USA) at concentration 2*10^10 pfu per sample was incubated with immobilized purified monoclonal antibodies (100µg per sample). Unbound phages were washed away. Antibodies with attached phages were eluted in 50 µl 0.1M glycine pH 3. Eluate was subsequently neutralized with 8µl 1M Tris pH 8. DNA sequencing library of eluted phages was prepared in three sets of PCR and sequenced using the Illumina Nextseq 550 system (Illumina, CA, USA). The reads derived from phage display samples underwent initial trimming and unique 12 amino acid sequences were analyzed. The sequences were analyzed with Hammock software developed previously (37), which employs a hidden Markov model-based clustering algorithm. The output was then visualized as a sequence logo using the WebLogo generator (38).

## 3 Results

### 3.1 Monoclonal antibodies against canine PD-1

Monoclonal antibodies (mAbs) against the canine PD-1 protein were generated by Moravian-Biotechnology Ltd. (Brno, Czech Republic). We chose two clones for further characterization, designated PD1-1.1 (isotype IgG2a) and PD1-2.1 (isotype IgG2b). Both antibodies possessed kappa light chains.

### 3.2 The antibodies detect cPD-1 in Western Blot

**Figure 1:**
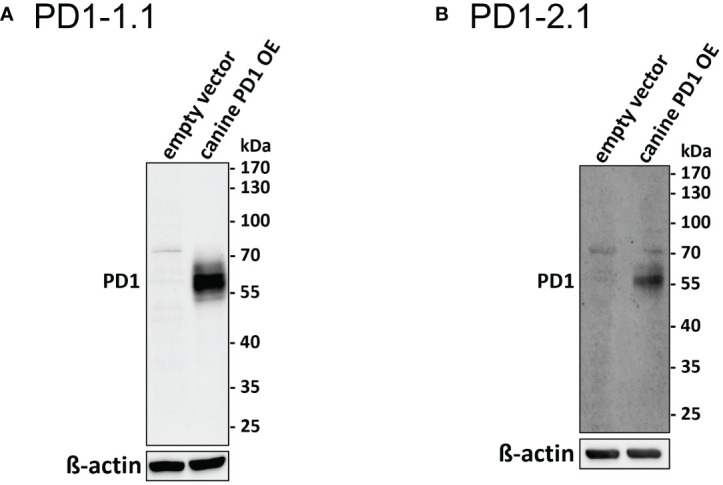
Western blot analysis of PD1-1.1 and PD1-2.1 mAbs binding to cPD-1. U2OS cells were transfected with a cPD-1 encoding plasmid or an empty vector as a negative control. **(A)** (PD1-1.1) and **(B)** (PD1-2.1) show distinct bands at ~60kDa in cPD-1 transfected cells, confirming specific binding of both antibodies to their target. No bands are observed in the negative controls. Below each blot, a cropped image of ß-actin bands from the same membrane (following stripping and re-probing) is presented as a loading control. Molecular weight markers are displayed on the right side of each blot. A faint additional band around 70 kDa may be due to non-specific reactivity of our anti-canine or secondary anti-mouse antibodies with human proteins. This artifact is absent in HEK cells (**Figure 2**; **Supplementary Figure S2**). Uncropped ß-actin blots are provided in **Supplementary Figure S1**.

To confirm the binding of cPD-1 by PD1-1.1 and PD1-2.1 mAbs, we conducted a Western Blot analysis using a lysate of a human osteosarcoma U2OS cell line transfected with an expression plasmid encoding canine PD-1. Lysate from an empty vector-transfected cell line was used as negative control. Both antibodies successfully detected the target protein, evidenced by a distinct band at approximately 60kDa (**Figure 1**). While the predicted molecular weight of cPD-1 is approximately 32kDa, it is known to migrate at around 60kDa, which is attributed to protein glycosylation (25). This band was absent in the negative control.

### 3.3 The antibodies are not cross reacting between human and dog proteins

**Figure 2:**
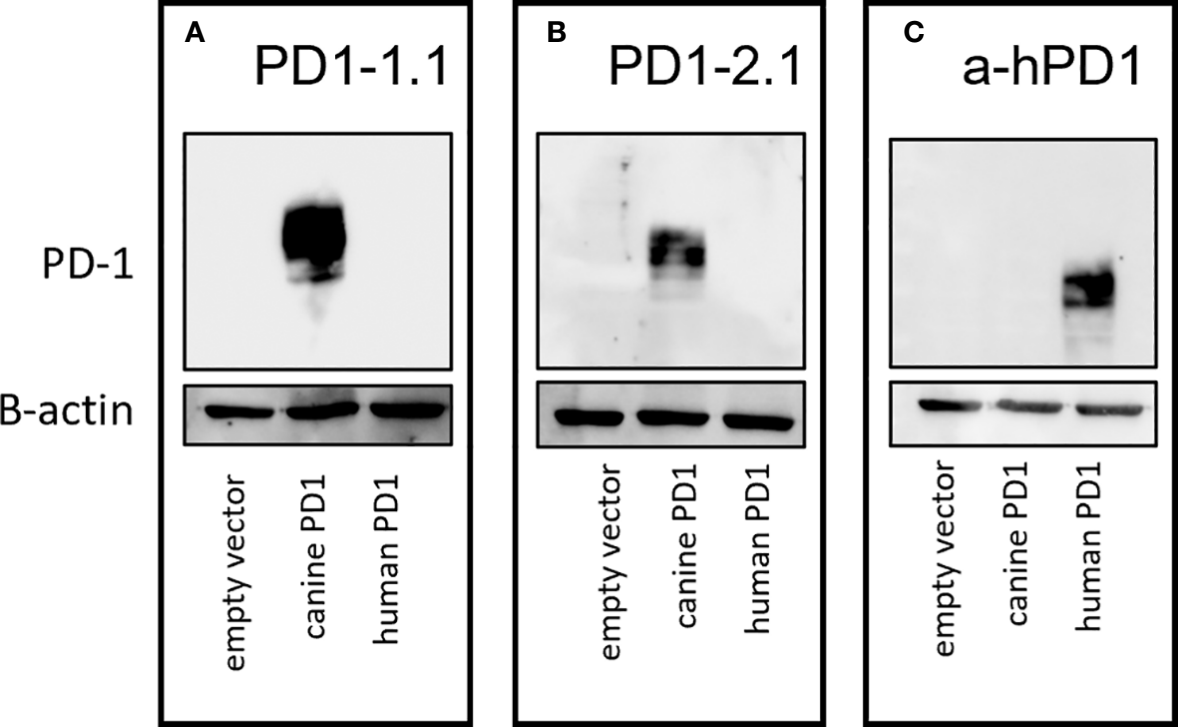
Specificity of the developed antibodies against canine and human PD-1. Western blot analysis of HEK293 cells transfected with plasmids encoding either canine or human PD-1. **(A)** PD1-1.1 antibody specifically detected canine PD-1 but not human PD-1. **(B)** PD1-2.1 antibody also selectively recognized canine PD-1 without cross-reactivity to human PD-1. **(C)** A commercially available anti-human PD-1 antibody served as a positive control, showing exclusive binding to human PD-1. Below each blot, a cropped image of ß-actin bands from the same membrane (following stripping and re-probing) is presented as a loading control. Original, uncropped images are provided in **Supplementary Figure S2**.

Some antibodies that target clinically important proteins cross-react between human and canine organisms. Several - including human anti-cancer antibodies - can bind to the canine versions of their target proteins and retain some functionality (39, 40). To evaluate the specificity of our antibodies for canine PD-1, we transfected HEK293 cells with plasmids encoding either canine or human PD-1, prepared cell lysates and detected respective proteins using PD1-1.1 and PD1-2.1 in Western Blot. A commercially available anti-human PD-1 antibody served as a positive control for human PD-1 expression. As shown in **Figure 2** below, PD1-1.1 and PD1-2.1 recognized the overexpressed canine PD-1 but not the human PD-1, which, in contrast, was detected by the human-specific antibody. We therefore concluded that the developed antibodies are specific for canine PD-1 and likely do not bind to the conserved regions of the PD-1 amino acid sequence.

### 3.4 The antibodies detect rcPD-1 in ELISA

**Figure 3:**
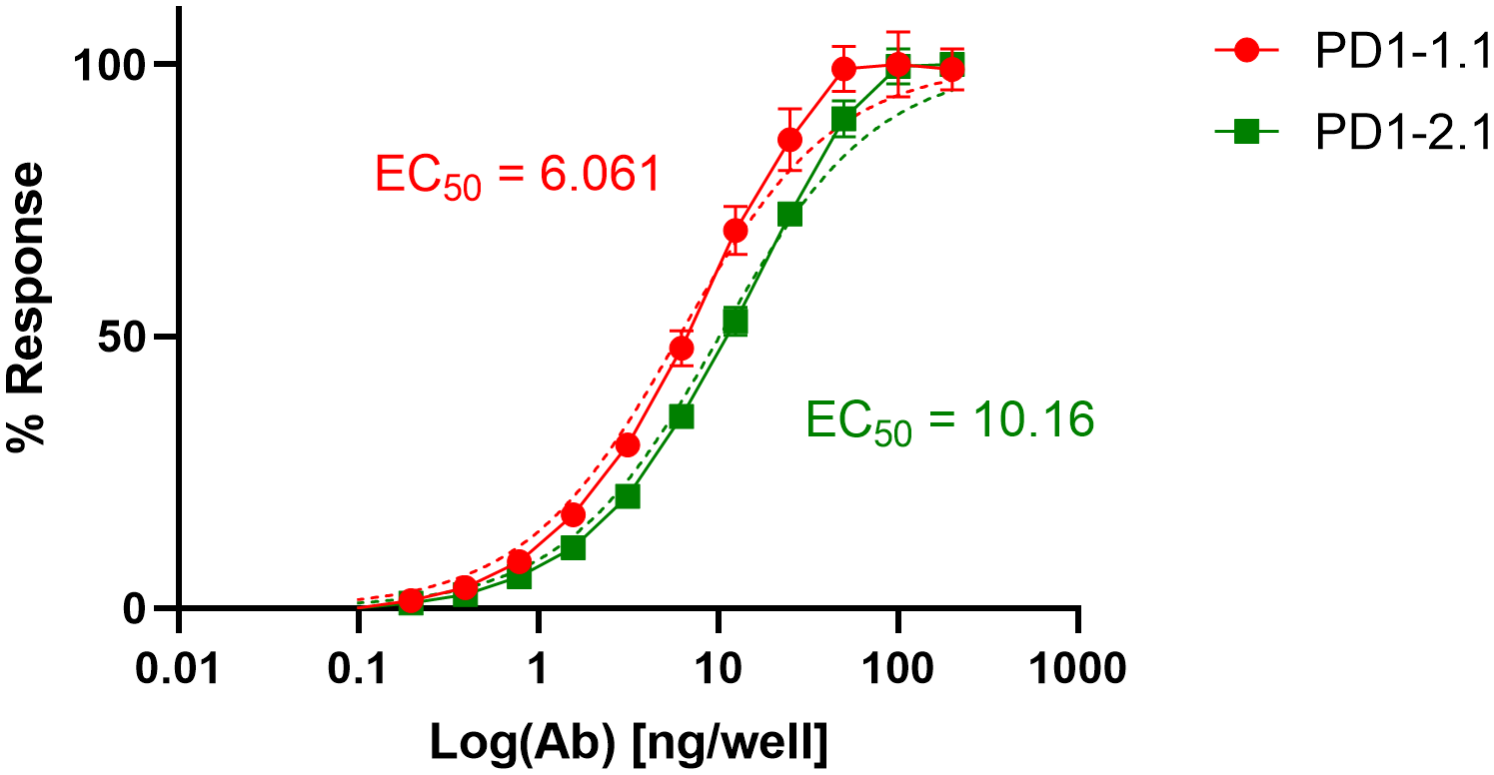
Binding curves of monoclonal antibodies PD1-1.1 and PD1-2.1 in ELISA against recombinant canine PD-1. The curves were plotted on a logarithmic scale and used to calculate EC50 values. PD1-1.1 showed a higher affinity compared to PD1-2.1. Error bars represent standard deviation from three replicates per datapoint.

Aiming to compare the two mAbs in a quantitative manner, we performed an ELISA. Dilutions of PD1-1.1 and PD1-2.1 mAbs were assayed on a plate coated with recombinant canine PD-1 (rcPD-1). When the results were visualized on a log scale, both antibodies presented characteristic sigmoid curves (**Figure 3**), which were used to calculate EC50 values. For the PD1-1.1, the EC50 value was determined to be 6.061 with a 95% confidence interval of 5.388 to 6.818. The R-squared value for the fit was 0.9836. For the PD1-2.1, the EC50 value was 10.16 with a 95% confidence interval of 9.236 to 11.18. The R-squared value for the fit was 0.9886. The lower EC50 value indicates that PD1-1.1 has a higher affinity for the target protein than PD1-2.1. Of note, the signal in ELISA was very high despite the target coating concentration in the lowest range of the common spectrum. While a high-sensitivity substrate was used to develop the results, this demonstrates very good performance of the tested antibodies in ELISA and possibly a high affinity to canine PD-1 protein in its native state.

### 3.5 The antibodies bind native canine PD-1 in flow cytometry

**Figure 4:**
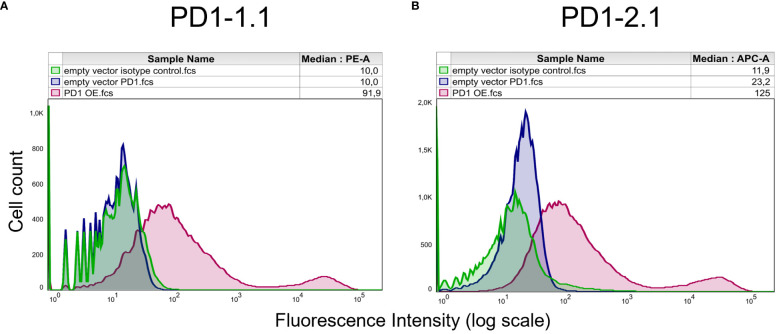
Flow cytometry analysis of PD1-1.1 and PD1-2.1 mAbs binding to cPD-1 expressed on live U2OS cells. U2OS cells were transfected with a cPD-1 encoding vector or an empty vector and then stained with PD1-1.1 **(A)** or, PD1-2.1 **(B)**, and an isotype control antibody. Each plot visualizes cells stained with a tested antibody (magenta), empty vector control (blue) and isotype control (green). **(A)** The median fluorescence intensity (MFI) for PD1-1.1 was 91.9 (PE-A channel). **(B)** The MFI for PD1-2.1 was 125 (APC-A channel). Both antibodies detected cPD-1 positive cells but not the negative control cells. The results indicate that both antibodies specifically recognize native cPD-1 expressed on live cells. Note: APC (allophycocyanin) and PE (phycoerythrin) are fluorescent dyes used for labeling antibodies. The ‘-A’ refers to the specific channel on the flow cytometer used to detect the fluorescence signal.

Next, we sought to assess the ability of our antibodies to bind cPD-1 in its most natural state, as expressed on live cells. To test this, we transfected U2OS cells with a cPD-1 encoding vector or an empty control vector. We stained the cells with PD1-1.1, PD1-2.1, or an isotype control antibody and analyzed them using flow cytometry (**Figure 4**). An isotype control antibody, a non-specific antibody of the same isotype as the primary antibody used in an experiment, serves as a negative control to account for non-specific binding, particularly in assays based on live cell. This control allows us to differentiate specific antibody-antigen interactions from background binding inherent to the antibody class. In this experiment, an isotype control antibody was used to stain cells transfected with the empty vector. Both PD1-1.1 and PD1-2.1 exhibited a shift in signal peaks for cells transfected with the PD-1 as compared to the control cells, indicating specific binding of PD-1. The peaks for PD-1 negative cells stained with PD1-1.1 and PD1-2.1 corresponded with the position of respective isotype control peaks, further indicating specific binding. Notably, PD1-2.1 displayed a higher median fluorescence intensity (MFI) than PD1-1.1, suggesting it may have superior sensitivity to PD-1 in the flow cytometry conditions.

### 3.6 The antibodies bind cPD-1 with high affinity in SPR

**Figure 5:**
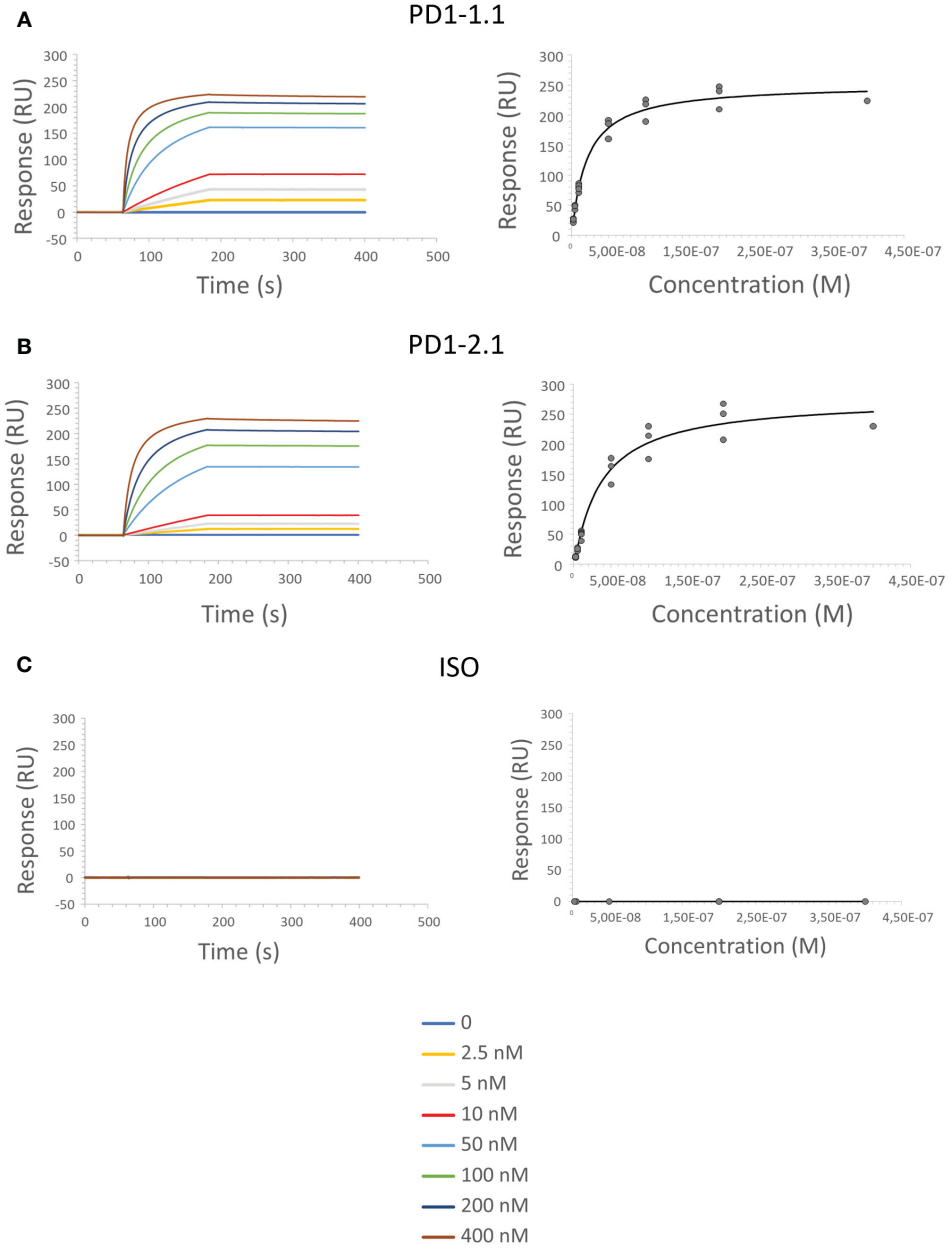
SPR sensorgrams show an increase in signal as the function of time and antibody concentration, indicating the binding between PD-1 immobilized on the sensor surface and the PD1-1.1 **(A)** PD1-2.1 **(B)** antibodies; no binding was observed for the isotype control antibody **(C)**.

Surface Plasmon Resonance (SPR) enables semi-absolute quantitation of protein-ligand interaction. While results always depend on the experimental setup, SPR is a golden standard for assessing the binding potential of therapeutic antibody candidates. We immobilized His-tagged rcPD-1 on dextran chips and assayed PD1-1.1, PD1-2.1 and an isotype control antibody (**Figure 5**). Based on the obtained curves, K_a_ (Association Constant), K_d_ (Dissociation Constant) and K_D_ (Equilibrium Dissociation Constant) were computed for each antibody (**Table 1**). KD is a measure of the overall affinity between two interacting molecules in equilibrium and has units of molarity (M). PD1-1.1 and PD1-2.1 displayed sub-nanomolar equilibrium dissociation constant. The isotype control generated no binding signal, thus validating the assay. The SPR analysis revealed the generated mAbs have a very high affinity to canine PD-1, sufficient for further development into therapeutics.

**Table 1:** Kinetic constants were calculated for each antibody from the obtained SPR data: association rate (Ka), dissociation rate (Kd) and equilibrium dissociation constant (KD); ISO, isotype control antibody; NA, not applicable (no binding detected).

	PD1-1.1	PD1-2.1	ISO
**K_a_ (1/Ms)**	3.37E+05 ( ± 3.12E+04)	1.75E+05 ( ± 1.91E+04)	NA
**K_d_ (1/s)**	4.93E-05 ( ± 1.5E-05)	2.36E-05 ( ± 1.43E-05)	NA
**K_D_ (M)**	1.5E-10 ( ± 6.16E-11)	1.42E-10 ( ± 1.04E-10)	NA

### 3.7 Both antibodies are PD-1/PD-L1 inhibitors in ELISA

**Figure 6:**
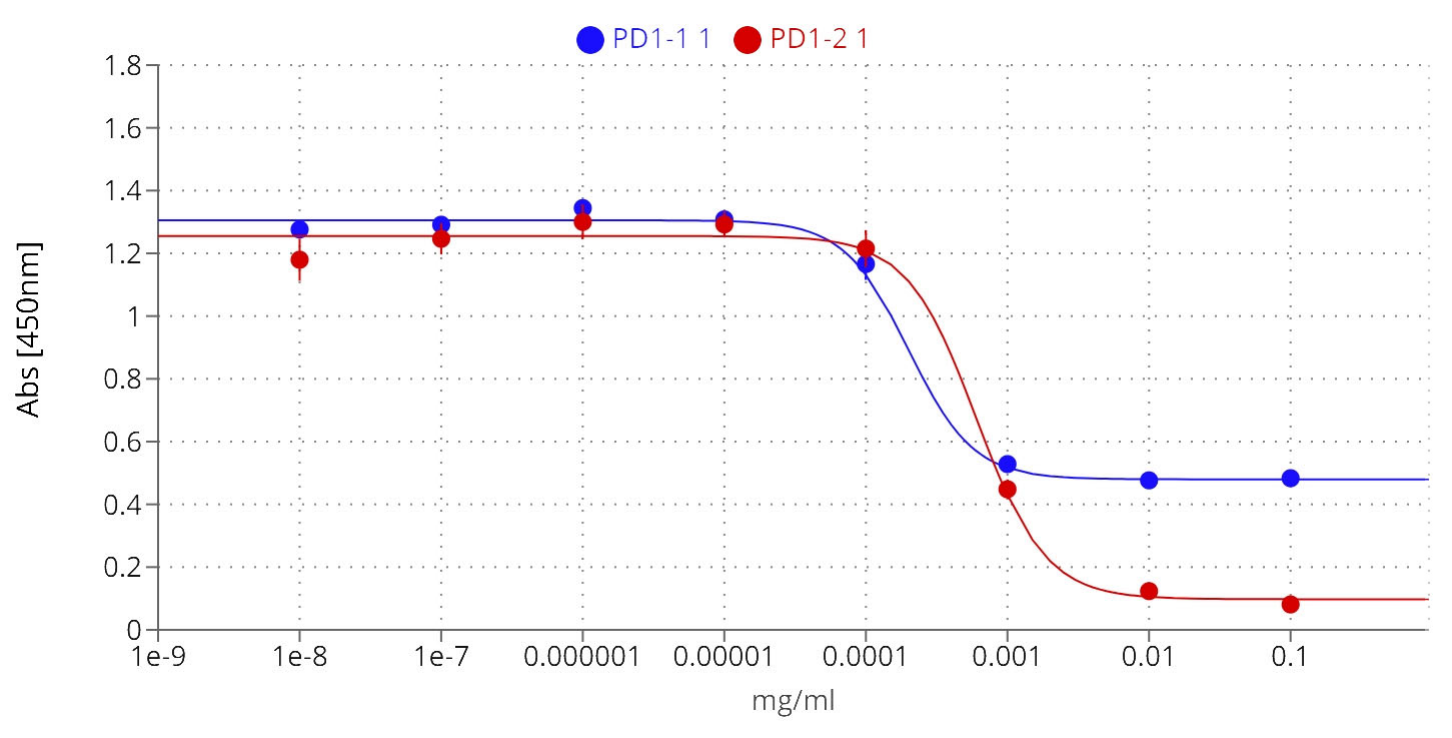
PD1-1.1 and PD1-2.1 both inhibit the PD-1/PD-L1 binding in ELISA, yet differ in their blocking capacity at the higher concentration, suggesting distinct binding sites or mechanisms; IC50 [PD1-1.1] = 0.0002, IC50 [PD1-2.1] = 0.0006; error bars represent standard error of the mean (SEM).

The efficacy of immune checkpoint inhibitors, unlike that of many other therapeutic antibodies, depends on their ability to disrupt the interaction between the IC receptor and its ligand of interest. To assess the therapeutic potential of PD1-1.1 and PD1-2.1, we needed to evaluate not only their binding to cPD-1, but also their capacity to prevent cPD-1 from binding to cPD-L1. To this end we used a competitive ELISA assay in which rcPD-1-His was immobilized on the plate and incubated with dilutions of the antibodies under investigation. After washing the plates, rcPD-L1-Fc was added, and its binding was subsequently detected with an anti-human HRP conjugate (**Figure 6**). In controls without the tested antibodies, PD-1-PD-L1 binding was entirely unblocked, resulting in the highest signal. Conversely, a reduction in the signal corresponded to successful inhibition of the PD-1-PD-L1 interaction.

Both PD1-1.1 and PD1-2.1 demonstrated their capability to disrupt the PD-1/PDL-1 interaction. The calculated IC50 value, based on four-parameter logistic regression, was lower for PD1-1.1 (0.0002) compared to PD1-2.1 (0.0006), indicating a higher potency at mid-range concentrations. However, at higher antibody concentrations, only PD1-2.1 exhibited a nearly complete blockade. This was evidenced by a significantly lower ‘bottom’ plateau in the ELISA curve (~0.1) compared to PD1-1.1 (~0.5). These results suggest that while PD1-1.1 might be slightly more potent at moderate concentrations, PD1-2.1 is far more effective at higher, therapeutically meaningful concentrations of 10-100µg/mL that resemble serum antibody levels in patients undergoing immunotherapy (41–43). Such differences could stem from the antibodies targeting distinct epitopes on cPD-1 or their specific binding orientation. These findings underscore the importance of considering affinity in the wider context of the specific binding mechanisms.

### 3.8 PD1-1.1 but not PD1-2.1 is suitable for cPD-1 detection in IHC

**Figure 7:**
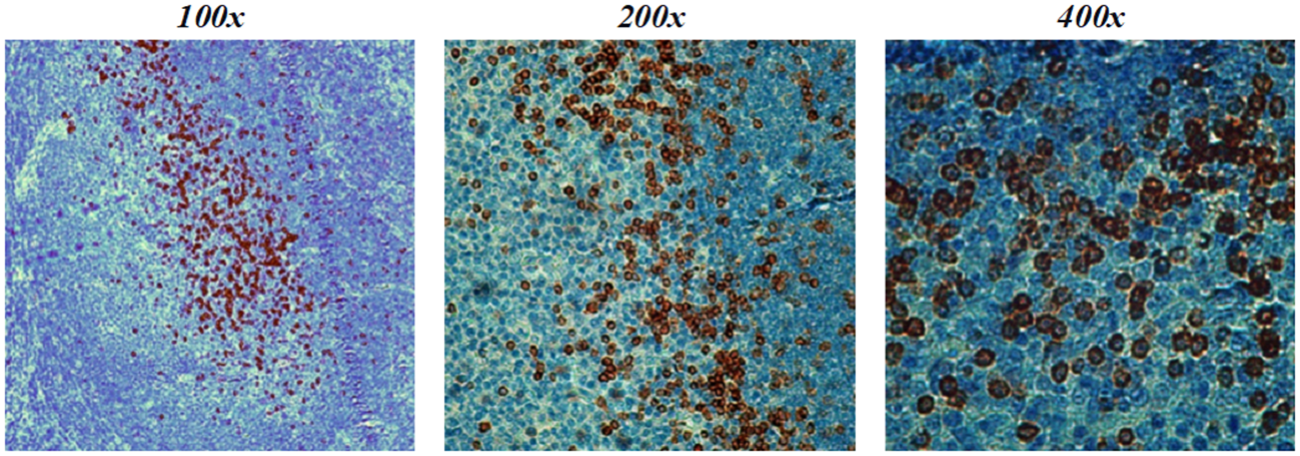
PD1-1.1 detects cPD-1 positive cell clusters in a canine tonsil tissue; magnification is 100x-400x and brown color marks the positively stained cells.

One of the clinically important molecular assays is the immunohistochemical (IHC) staining of cancer-affected tissues. We performed IHC staining of a tonsil lymphoid tissue sample with both antibodies and found that PD1-1.1 stained cell clusters with natural cPD-1 expression (**Figure 7**). Meanwhile, staining with PD1-2.1 could not be optimized to generate replicable results, hence we concluded only PD1-1.1 is suitable for detection of cPD-1 in IHC. In fact, we have successfully used the PD1-1.1 for an IHC analysis of clinical samples in a prior publication (26).

### 3.9 The antibodies detect cPD-1+ cells in immunocytochemistry

**Figure 8:**
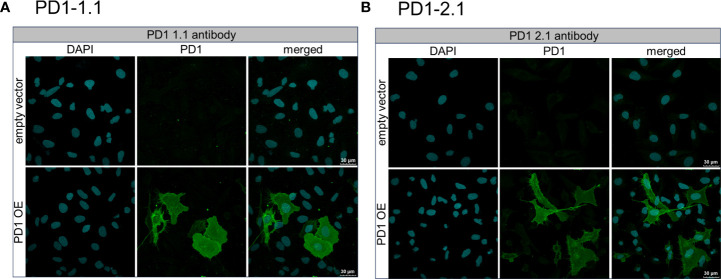
Immunofluorescent staining of U2OS cells to assess the binding specificity of PD1-1.1 and PD1-2.1 antibodies. U2OS cells with stable PD-1 overexpression and those transfected with an empty vector were incubated with either PD1-1.1 **(A)** or PD1-2.1 **(B)** antibodies. Bound antibodies were detected with fluorescently labeled secondary antibody (green), and the cell nuclei were stained with DAPI (blue). Each panel consists of two rows corresponding to cells transfected with an empty vector (top row) and cells with PD-1 overexpression (bottom row). The three columns display images of DAPI-stained nuclei (left), antibody staining (middle), and merged images (right). The green signal is exclusively present in PD-1 overexpressing cells, indicating the binding specificity of both antibodies to PD-1.

To investigate the binding of the PD1-1.1 and PD1-2.1 antibodies to PD-1+ cells in detail, we performed immunocytochemistry, an immunofluorescent (IF) cell staining technique, using U2OS cells with a stable cPD-1 overexpression and those transfected with an empty vector. We incubated these cells with either PD1-1.1 or PD1-2.1. Next we detected the bound antibodies with a fluorescently labeled secondary antibody and observed the fixed cells under a confocal microscope. The results of the immunofluorescent staining were displayed on **Figure 8**. For both PD1-1.1 (8A) and PD1-2.1 (8B), only the cells transfected with canine PD-1 generated a strong green light signal, indicating that they bound the antibodies. This result demonstrated the binding specificity of both antibodies and their suitability for IF staining experiments.

### 3.10 Modeling the cPD-1 epitope for PD1-1.1 and PD1-2.1

**Figure 9:**
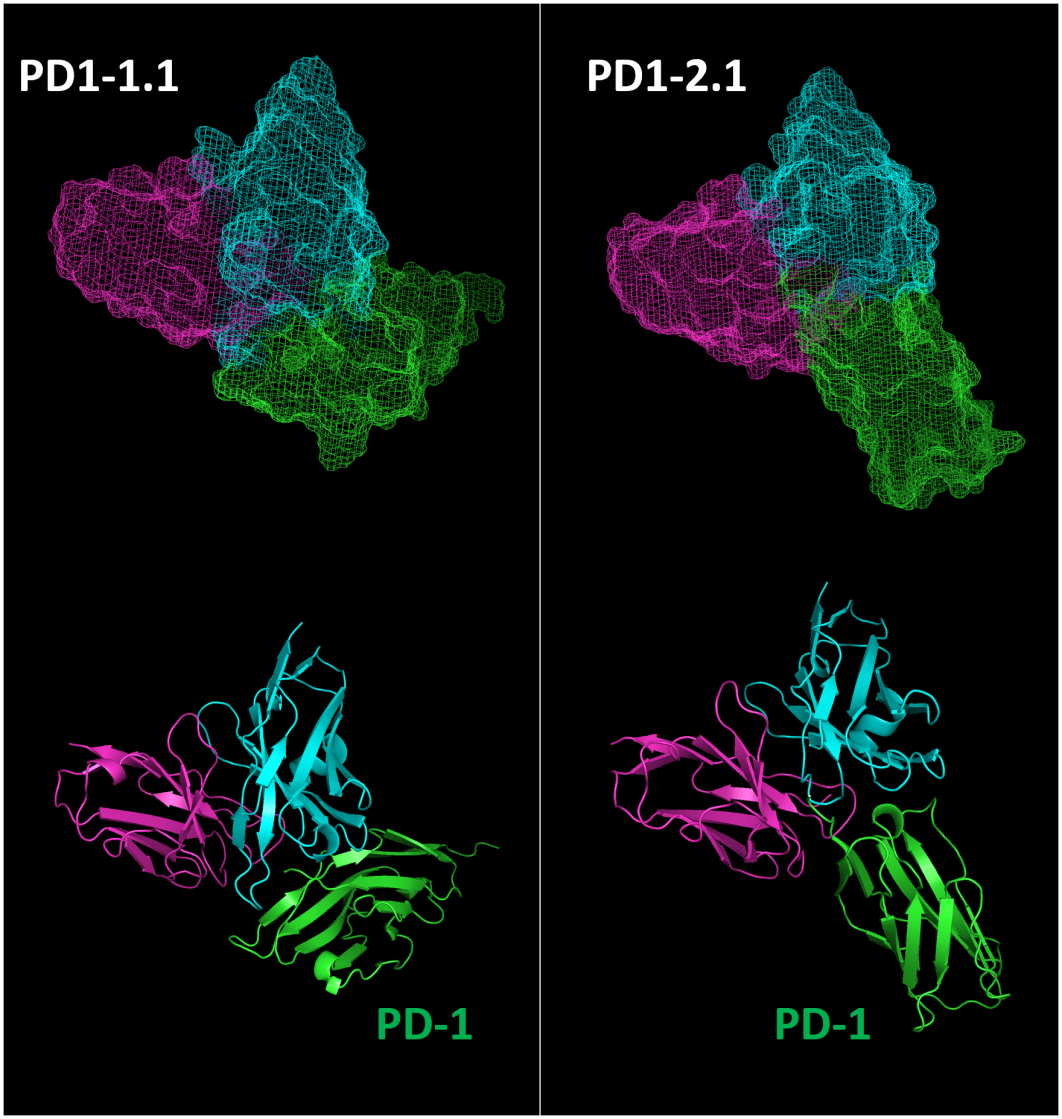
Top binding poses of PD1-1.1 and PD1-2.1 were identified and visualized with ‘mesh’ and ‘cartoon’ projections. Cyan – heavy chain, magenta – light chain, green – canine PD-1.

The two antibodies displayed very similar affinity by SPR, yet different PD-1/PD-L1 blocking characteristics and varying performance in other assays. To delineate the respective epitopes of the two antibodies, we initially employed various epitope prediction tools such as Discotope, BepiPred, AbAdapt, EpiPred, and IEDB Epitope Prediction. However, they were not able to narrow down the range of possible epitopes. This led us to develop a more targeted approach. We modeled the structures of the PD1-1.1 and PD1-2.1 variable domains as well as the structure of the canine PD-1. Subsequently, we performed docking of variable domains to canine PD-1. For each antibody we chose the top most probable binding model (**Figure 9**).

**Figure 10:**
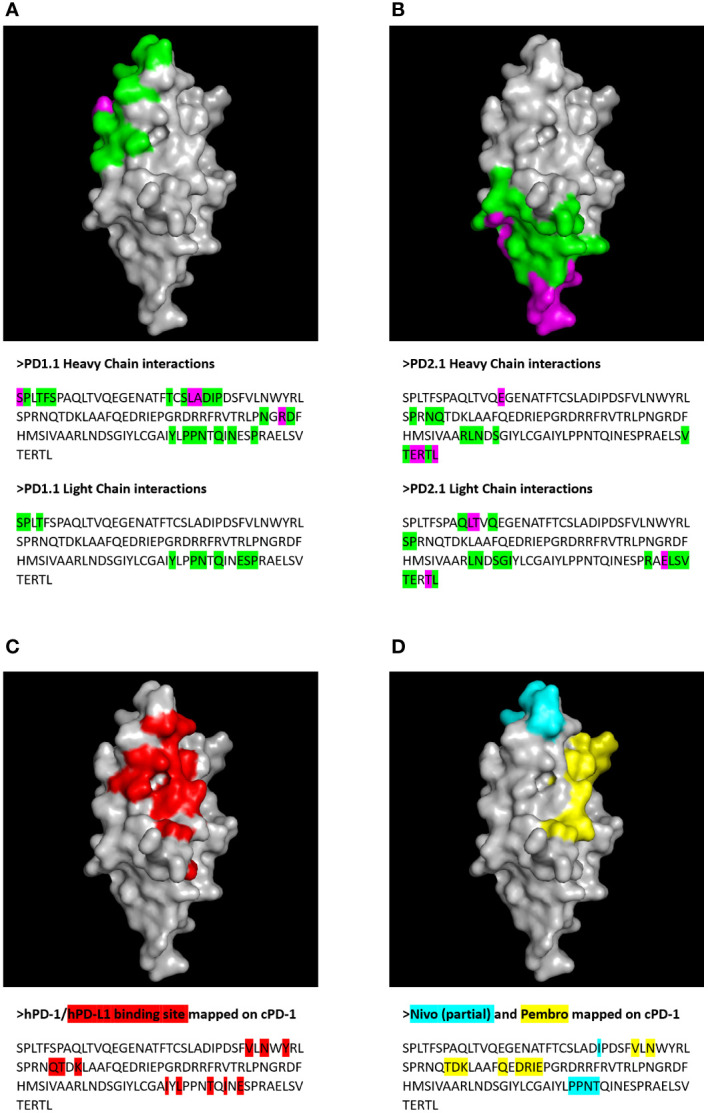
Putative epitopes of PD1-1.1 and PD1-2.1 on canine PD-1 (cPD-1) were identified by molecular modeling and docking. Receptor residues participating in the protein-protein interaction were visualized on the cPD-1 structure and sequence for PD1-1.1 **(A)** and PD1-2.1 **(B)**. Green – residues participating in the interface; magenta – interface residues contributing hydrogen/disulphide bonds, salt bridges or covalent links. For comparison, the cPD-1 regions corresponding to the human PD-1 interaction sites for PD-L1 (44), Nivolumab (Nivo (45)) and Pembrolizumab (Pembro (46) were marked with red **(C)**, cyan and yellow **(D)**, respectively.

Next, we extracted residue positions that formed the antibody-target interface. These putative PD-1 epitopes were mapped on to the sequence and structure of the PD-1 protein (**Figure 10**). The antibody residues participating in the interface belonged to CDR regions. The cPD-1 residues participating in the interface differed between the PD1-1.1 and PD1-2.1 antibodies, suggesting distinct epitopes. Additionally, the sites of human PD-1 interaction with its ligand and therapeutic antibodies Pembrolizumab and Nivolumab (44–46) were mapped onto the homologous parts of the canine PD-1 structure (**Figure 10**). These sites were distinct, yet slightly overlapping with our putative epitopes.

### 3.11 Epitope mapping by phage-peptide display

**Figure 11:**
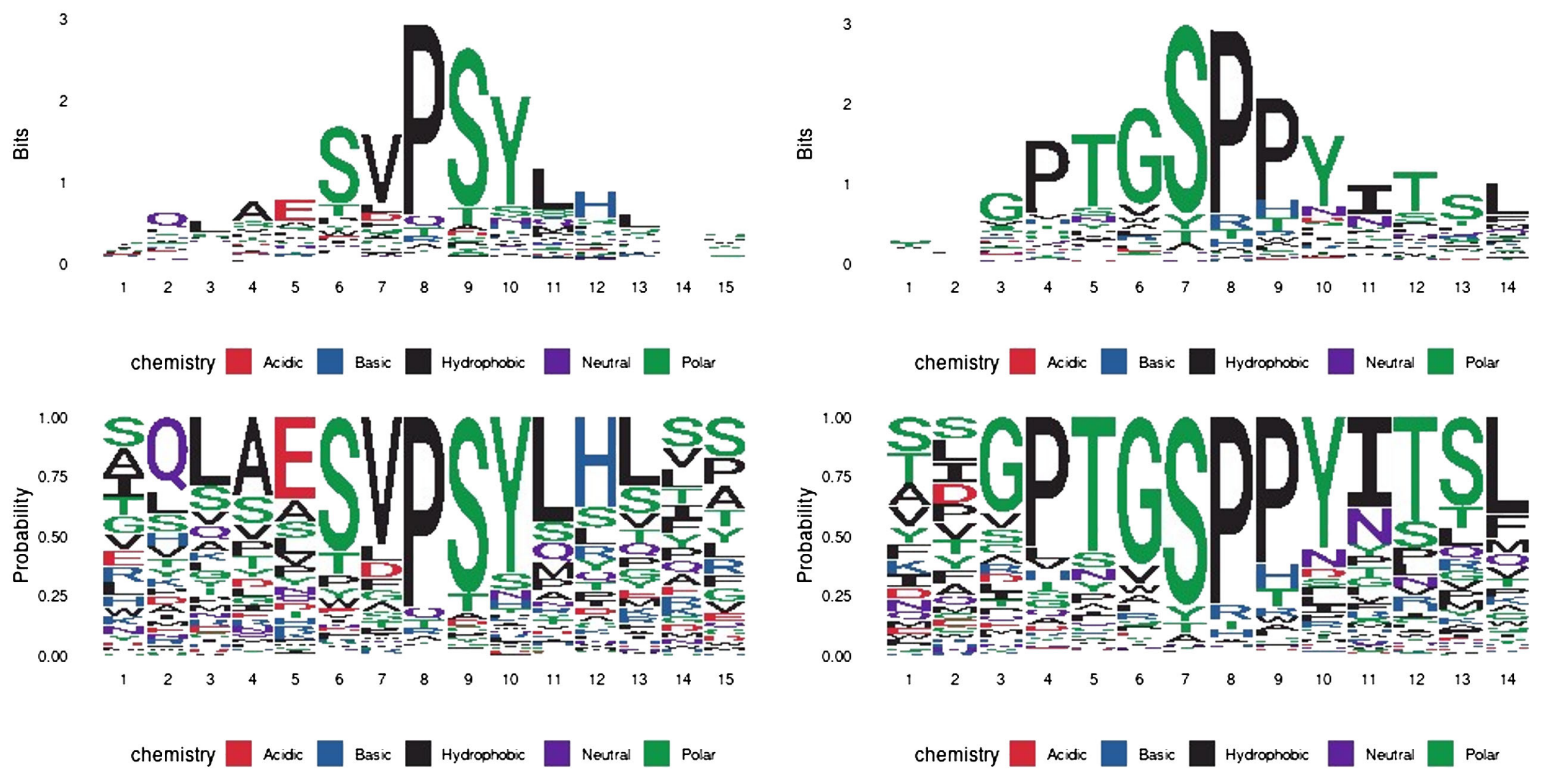
Epitope mapping by peptide display and HAMMOCK analysis. The sequence logos represent residue conservation in the identified peptide sequence clusters. The values are presented in bit units or as a residue probability. The most conserved residues form mimotope motifs: ‘SVPSY’ and ‘PTGSPPY’ for PD1-1.1 and PD1-2.1, respectively.

We next carried out a peptide phage display to biologically determine antibody-binding mimotopes from a library of peptides and assess their homology to the canine PD-1 sequence. In this experiment, we incubated the peptide-presenting phages with each antibody of interest, immobilized. We eluted the bound phages, sequenced their DNA and obtained 12-amino acid sequences which were subsequently aligned with the cPD-1 protein sequence. Additionally, we used Hammock software (37), designed to identify binding motifs via a clustering algorithm. This yielded two potential epitope motifs: SVPSY and PTGSPPY for PD1-1.1 and PD1-2.1, respectively (**Figure 11**). Using Clustal Omega, we found no strong alignment of these motifs to the extracellular part of the canine PD-1 protein sequence. Nevertheless, these results suggest the two antibodies bind different protein motifs, hinting once again at distinct epitopes.

## 4 Discussion

Here, we introduce two monoclonal antibodies (mAbs) against canine PD-1 - PD1-1.1 and PD1-2.1 - to address an unmet need for immune checkpoint blockers in canine oncology. We join the pioneering efforts by Coy (17) and Nemoto (25, 47) as the third group to report such antibodies, yet the first to conduct a comprehensive evaluation across the key molecular assays.

Uniquely, by utilizing surface plasmon resonance (SPR), we found that both antibodies have high affinity for PD-1, with sub-nanomolar KD values. This characteristic nominates them as suitable candidates for therapeutic development. To evaluate their PD-1-inhibitory dynamics, we developed a novel competitive ELISA assay. Here, PD1-2.1 demonstrated superior blocking activity over PD1-1.1, despite comparable target affinity of both mAbs in SPR. Interestingly, the mAbs exhibited similar IC50 values within lower concentration ranges, but at the higher range PD1-1.1 reached a plateau. Meanwhile, PD1-2.1 continued to decrease the signal, achieving a nearly complete PD-1 blockade at a concentration mimicking therapeutic antibody levels in blood. The superior blocking activity of PD1-2.1 stands in contrast with its inferior binding in a classic PD-1 binding ELISA. This discrepancy underscores the importance of evaluating functional capabilities in tandem with the affinity metrics.

Both our mAbs recognized canine but not human PD-1 in WB. Further, they were functional and specific in flow cytometry (FC), and immunocytochemistry (IC) assays. However, only PD1-1.1 was effective in immunohistochemical staining (IHC). The functional divergence between the two mAbs may be attributed to different epitopes, possibly involving post-translational modifications (48). The PD1-2.1 epitope is likely more conformational in nature or less stable in denaturing conditions.

In initial Western blots against naturally expressed and recombinant PD-1 both our antibodies demonstrated weak signal. To overcome this limitation, in subsequent WB and IC experiments we utilized cell lines overexpressing canine PD-1 (U2OS and HEK293), similarly as Coy (CHO cells) and Nemoto (NRK cells). These observations suggest that our antibodies target conformational epitopes. This feature renders antibodies suitable for therapeutic applications at the cost of weaker performance in molecular assays involving denaturation.

Indeed, an exploratory molecular docking experiment predicted distinct, non-continuous epitopes. The difference between PD1-1.1 and PD1-2.1 binding sites on canine PD-1 was further supported by our mimotope analysis based on peptide phage display. The identified mimotopes did not however exhibit homology to the extracellular portion of the PD-1 protein and thus did not reveal the exact epitopes.

Further, we attempted to evaluate the ability of our mAbs to activate T-cells in an assay based on peripheral blood mononuclear cells (PBMCs). However, we identified several shortcomings of the assay, precluding its meaningful interpretation. Details of the assay, detected issues and extended commentary on its weakness are included in the supplementary section. Briefly, in our view, the assay is too dependent on the blood donor medical histories, inter-donor variability, plagued by low statistical power, inconsistent results and artifacts commonly seen in the literature.

Previously, Coy et al. described two mAbs against PD-1, which were functional in WB, FC, and additionally detected CD4+ and CD8+ T-cells from healthy dog blood in FC (17). Nemoto et al. also validated two anti-PD-1 mAbs in FC against PD-1 overexpressed in a cell line and induced in PBMCs (25). They also detected PD-1 in WB. Of note, in their work, the WB results showed bold secondary bands in negative control cells and their FC-based assays yielded inconclusive results, likely attributable to the specific experimental setup. Functionality in IHC or IC was not tested by the other groups.

The unique strength of the work by Coy et al. was their dis-inhibition test of one mAb, where ConA-activated PBMCs were ‘inhibited’ with either recombinant cPD-L1 or cPD-L1-overexpressing cells (17). In the latter assay, IFN-γ production by PBMCs was unequivocally inhibited and one of the mAbs partially reversed this inhibition, with a statistically significant effect. Impressively, they have repeated this experiment for both mAbs while using tumor explant cultures as PBMC suppressors. Here, both antibodies reversed the tumor’s inhibitory impact. They have additionally demonstrated increased proliferation and IFN-γ secretion in tumor-infiltrating lymphocytes extracted from cancer tissues and treated with the PD-1 antibodies.

In an important contribution, Igase et al., building upon the foundational work of Nemoto et al., advanced the field by taking their antibody characterization into a clinical setting (47). In collaboration with Nexvet and Zenoaq companies, they re-engineered 4F12-E6 - a previously characterized monoclonal antibody - into a chimeric and a fully caninized form. This antibody exhibited promising results in flow cytometry and PBMC IFN-γ secretion assays, despite some anomalies and the lack of isotype control in the latter. Crucially, their work culminated in an animal trial. The treatment led to a statistically significant decrease in overall survival when compared to historical controls. Although this bold study suffered from methodological issues and the interpretation of trial results invites careful scrutiny, it stands as an important attempt at translating lab bench developments to the bedside. Following this path, we are progressing toward the caninization of our antibodies, a crucial step for our ultimate goal of clinical trials.

Our antibodies against canine PD-1 stand out as comprehensively characterized and uniquely versatile across diverse molecular assays. PD1-1.1 excels in diagnostic applications, whereas PD1-2.1 shows greater promise as a potential therapeutic. Further research is warranted to validate the potential of developed antibodies in both domains.

## 5 Future perspectives

### 5.1 Receptor or ligand - which one to block?

The sequence of PD-L1 - the prevalent PD-1 ligand - is more conserved than the PD-1 sequence (76% *vs* 66% identity, respectively). This corroborates the fact human anti-PD-L1 therapeutics Avelumab and Atezolizumab have been found to block canine PD-1 - PD-L1 interaction (39). While targeting PD-1 may be more therapeutically effective in some clinical settings (49), targeting PD-L1 may be associated with fewer adverse reactions (50). Importantly, both PD-1 ligands have been found to also engage other receptors (51, 52). Consequently, the biological impact of ligand blockade is related to modified signaling downstream of multiple receptors, rather than just PD-1.

On the other hand, a recent study in the field of autoimmune alleviation has illuminated a new mechanism: blocking the CD80 receptor can effectively redirect its ligand, PD-L1, to interact more preferentially with its alternative receptor, PD-1 (53). This process amplifies the agonistic effect on PD-1. Extrapolating from this, it is plausible to hypothesize that inhibiting PD-1 could conversely lead to a heightened agonistic effect on CD80. Furthermore, this process could possibly influence the activation of hitherto unidentified receptors for the PD-L1 and PD-L2 ligands. This potential for undesired receptor activation may underlie the phenomenon of hyperprogression observed in patients undergoing ICB immunotherapy.

The biological and clinical differences resulting from receptor and ligand blockade remain to be untangled and fully understood. To complete the PD-1 checkpoint toolkit, we are developing canine anti-PD-L1 mAbs.

### 5.2 The importance of PD-L2

In this study, we analyzed the blocking property of antibodies regarding the PD-1/PD-L1 interaction, while omitting the second known PD-1 ligand: PD-L2. This is a common practice, since PD-L2 expression had long been believed to be restricted to cytokine-stimulated macrophages and dendritic cells, and to remain insignificant (54). Since the ICI founding idea was to shield tumor-infiltrating lymphocytes from the cancer-expressed IC ligands, PD-L2 seemed irrelevant. However, light has been shed on the importance of immune checkpoint interactions between immune cell subtypes such as regulatory T-cells (TREGs), tumor-associated macrophages (TAMs), dendritic cells (DCs) and cytotoxic T-lymphocytes (55–57). Additionally, it was demonstrated that PD-L2 is expressed in stromal, immune and tumor cells, may bind PD-1 with higher affinity than PD-L1, and constitutes an essential immunotherapy target (58–60). Despite that, PD-L2 remains largely outside the research spotlight. In humans PD-L2 is known to bind PD-1 through a different mechanism than PD-L1 does, potentially making it affected differently by the IC inhibitors (61). Hence, the conclusions of this study cannot be extrapolated to PD1/PD-L2 interactions. The inhibition of PD-1/PD-L1 axis should not be thought of as a general PD-1 blockade in the context of this, and further studies.

### 5.3 Novel immune checkpoint targets

Finally, research into immune checkpoints is no longer limited to the PD-1 receptor and T-cells (62, 63). Questions arise about the multi-layer network of interactions between all ICs and all immune cell types. Recently, it has become apparent that alternative splicing of IC proteins adds to the already complex picture (64–67). We predict that monoclonal antibodies specific to IC splice variants and post-translationally modified forms will emerge as a new, more targeted generation of ICI therapeutics (48, 68).

## Data availability statement

The original contributions presented in the study are publicly available. This data can be found here: NCBI SRA repository, PRJNA1106919 (samples SAMN41153794-SAMN41153797).

## Ethics statement

Ethical approval was not required for the studies on humans in accordance with the local legislation and institutional requirements because only commercially available established cell lines were used. The animal study was approved by Veterinary Ethics Research Committee (Institutional Care and Use Committee; project number 96/21) at The Royal (Dick) School of Veterinary Studies, University of Edinburgh. The study was conducted in accordance with the local legislation and institutional requirements.

## Author contributions

MK: Conceptualization, Data curation, Formal analysis, Funding acquisition, Investigation, Methodology, Project administration, Visualization, Writing – original draft, Writing – review & editing. KD: Data curation, Investigation, Methodology, Validation, Visualization, Writing – original draft, Writing – review & editing. KW: Data curation, Investigation, Methodology, Visualization, Writing – review & editing. VH: Investigation, Writing – review & editing. FZ: Investigation, Writing – review & editing. BV: Data curation, Formal analysis, Funding acquisition, Investigation, Methodology, Project administration, Resources, Supervision, Writing – original draft, Writing – review & editing. JA: Funding acquisition, Resources, Writing – review & editing. TH: Conceptualization, Funding acquisition, Methodology, Resources, Visualization, Writing – review & editing. MP: Conceptualization, Funding acquisition, Investigation, Methodology, Project administration, Resources, Supervision, Writing – review & editing.
