# Supplementary material for: Comparative characterization of two monoclonal antibodies targeting canine PD-1
Source: Front Immunol. 2024 May 8;15:1382576. doi: 10.3389/fimmu.2024.1382576 (PMC11110041; doi:10.3389/fimmu.2024.1382576)
Supplement: Supplementary file 1 [file DataSheet_1.pdf]

Supplementary Materials

Supplementary Figures

Figure S1. ß-actin blots corresponding to the results shown in Figure 1 are displayed without cropping.

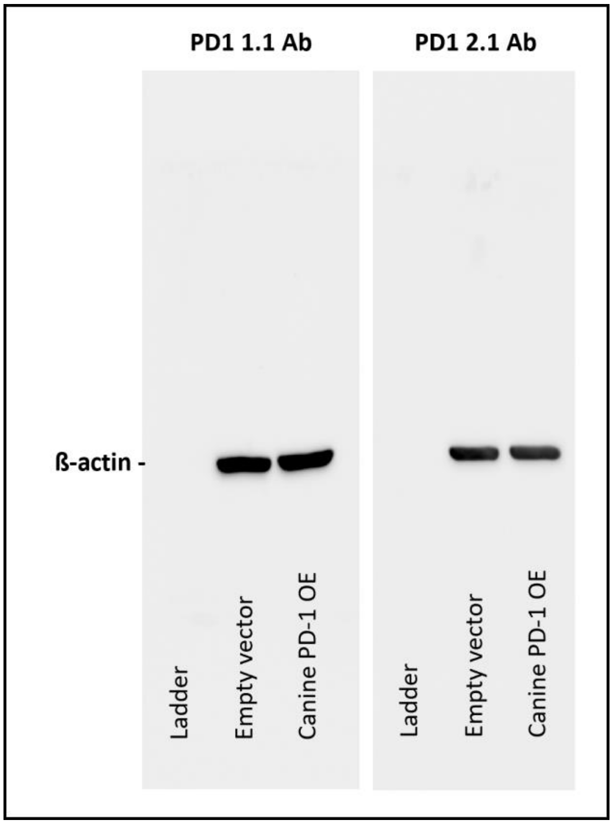

Figure S2. Western blots corresponding to the results shown in Figure 2 are displayed without cropping. These blots confirm the specificity of the PD1-1.1 (A), PD1-2.1 (B) to canine and not human PD-1, while the control anti-human PD-1 (C) antibody detected human and not canine PD-1 protein.

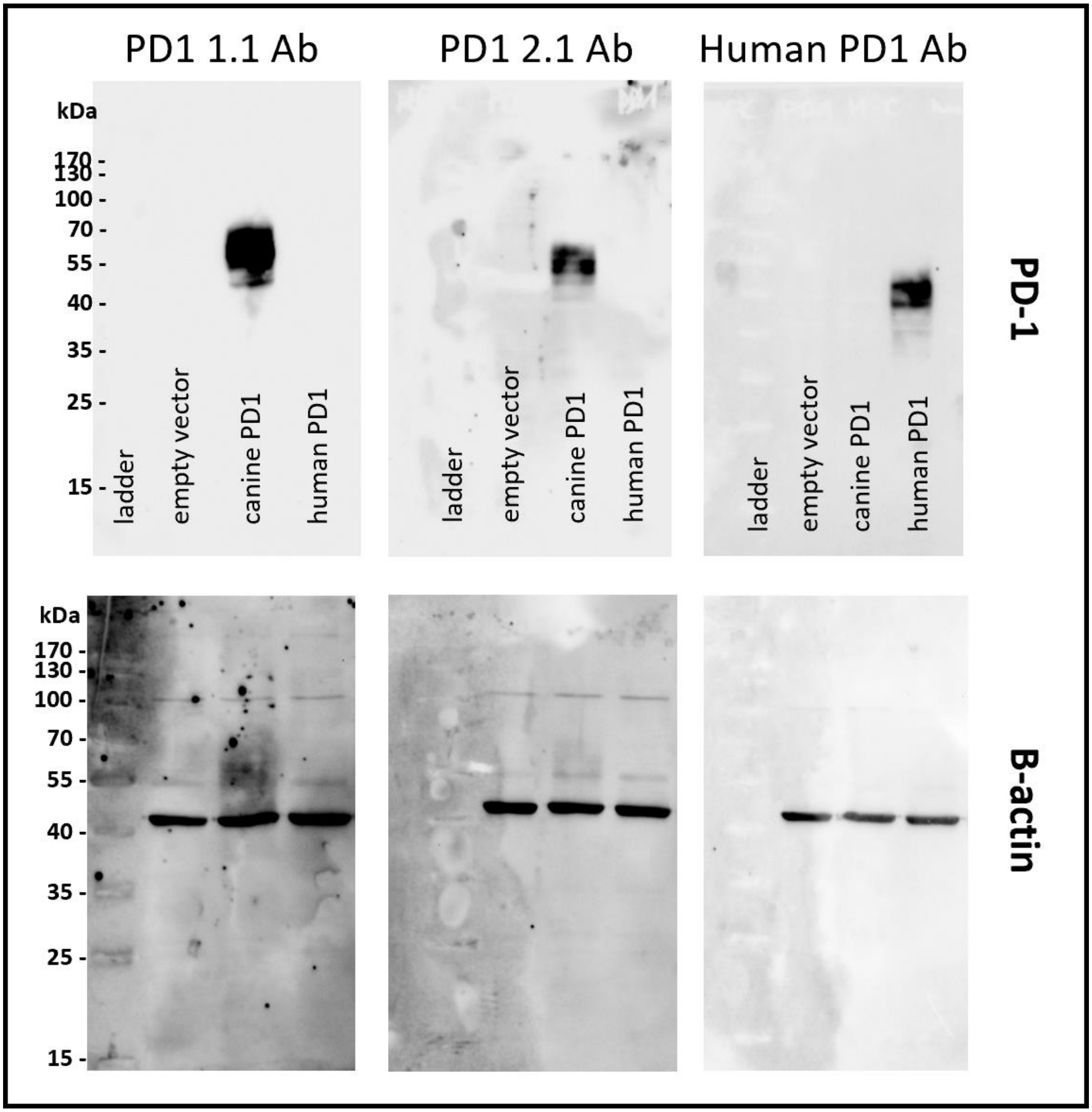

Supplementary results

PD1-1.1 but not PD1-2.1 disinhibits IFN-γ production in PBMCs

Having confirmed the PD-1/PD-L1 blocking properties of developed antibodies in ELISA, we strived to validate this functionality in a cell-based assay. To this end, we purified peripheral blood mononuclear cells (PBMC) fraction of whole blood from canine donors. PBMCs naturally include immune cell populations expressing PD-1 and PD-L1. Healthy T-cells which constitute a major part of PBMCs, when exposed to a T-cell receptor (TCR) stimulant Concanavalin A (ConA), which mimics a potent antigen, increase the expression of PD-1 and the secretion of cytokines such as Interferon-gamma (IFN-γ). At the same time, the contact between their PD-1 receptor and PD-L1 ligands of other PBMCs may decrease the rate of this process. The addition of PD-1 blocking antibodies prevents this decrease. Hence, the measurement of IFN-γ secretion from ConA-stimulated PBMCs is often considered a proxy for the PD-1 blocking capacity of an antibody. In our assay PBMCs obtained from healthy dogs were cultured, either unstimulated (baseline signal), stimulated to ConA (5µg/ml; positive control) or exposed to combinations of ConA with the tested antibodies or isotype control antibody (all antibodies - 10µg/ml) for 72h. After that the conditioned medium from cell culture was tested for the concentration of secreted IFN-γ by ELISA. We calculated the assay results for PBMCs from 5 dogs (Fig. S2A) and normalized them to the isotype control (Fig. S2B) to enable direct comparison. We calculated the mean result by averaging out the normalized results from five blood samples (Fig. S2C). All PBMC samples reacted to Concanavalin A stimulation (positive control), validating their use in the assay. As shown on Fig. S2C below, statistically significant differences between isotype control and antibody treatments have not been found, which can be partially attributed to a low statistical power of the test. This finding contrasts with the blocking observed in the competitive ELISA assay. Notably, PBMCs from one dog (#4 - dark green) reacted in the way that would be expected based on the prior ELISA, with signal higher for test antibodies than isotype control.

Figure S2: The ability of antibodies to block PD-1 was evaluated in a PBMC-based assay. Peripheral blood mononuclear cells (PBMC) were isolated from blood of canine donors and are a fraction high in immune cells. In the assay the blocking antibodies counteract inhibition of T-cells, resulting in increased secretion of IFN-γ. A) The mean and standard deviation based on duplicate measurements for each sample are presented. B) The data were normalized against the signal from the isotype control. C) The averaged data from PBMC samples taken from five dogs. IFN-γ secretion markedly increased upon exposure to PD1-1.1 and PD1-2.1 in PBMCs of one dog, but no statistically significant difference was found between isotype control and the characterized antibodies (Kruskal-Wallis and Dunn's post-hoc tests). Asterisks indicate statistical significance based on p-value thresholds: *$p < 0.05$, **$p < 0.01$, ***$p < 0.001$. IFN-γ - interferon gamma, Abs - absorbance, ConA - Concanavalin A.

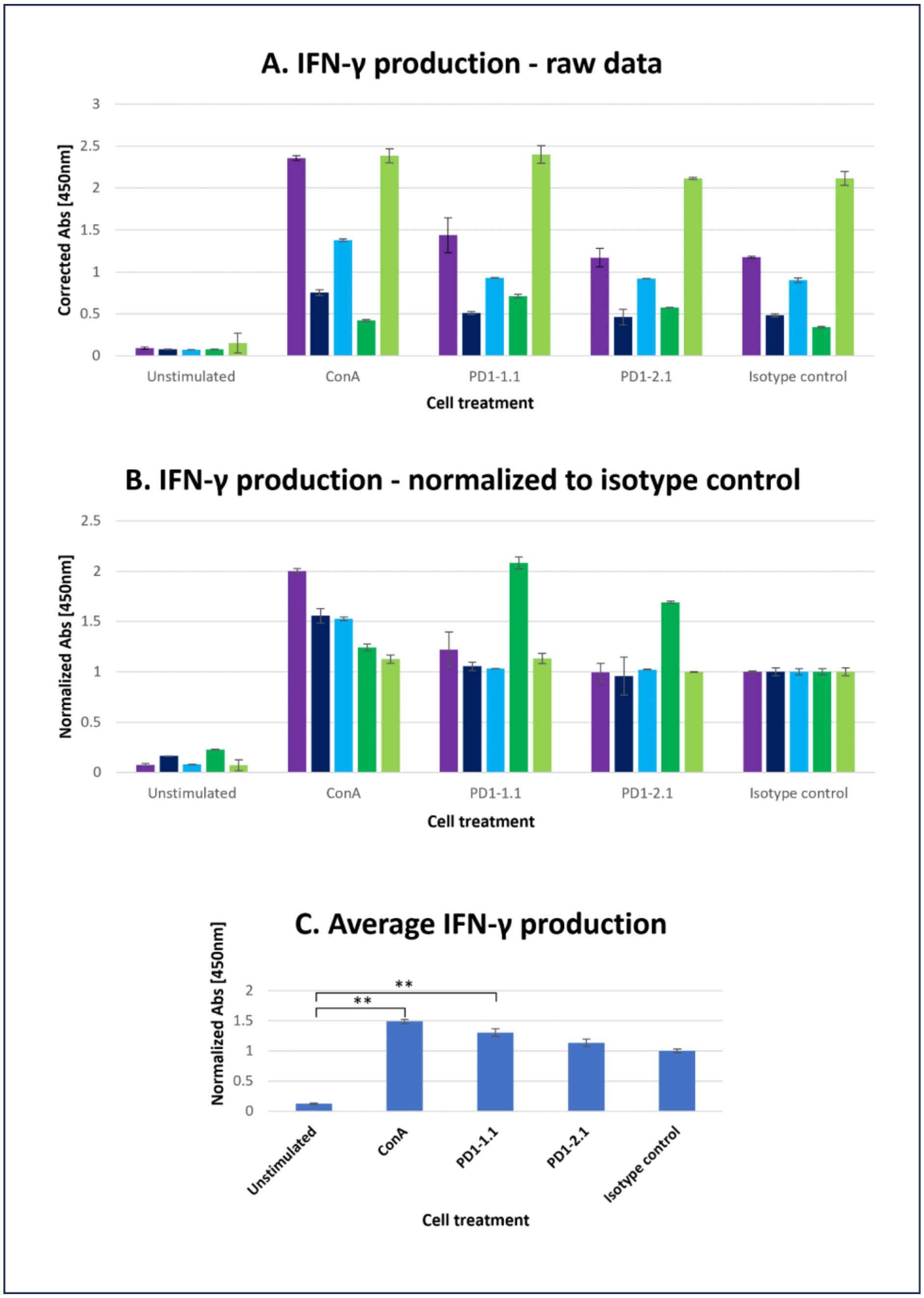

Supplementary methods

Tissue collection

Fresh blood was obtained from 8 dogs that were euthanized for reasons unrelated to this study. All tissue collection procedures were performed under the approval and guidance of the Veterinary Ethics Research Committee (Institutional Care and Use Committee; project number 96/21) at The Royal (Dick) School of Veterinary Studies, University of Edinburgh. Details regarding the blood donors can be found in Table 3.

Table S1. Details on the canine donors of blood used for PBMC extraction.

Number	Sex	Age	Breed	Health Status
1	male	1 year	Beagle	Healthy
2	female	11 months	Beagle	Healthy
3	male	1 year	Beagle	Healthy
4	male	10 months	Beagle	Healthy
5	female	11 months	Beagle	Healthy
6	male	14 months	Beagle	Healthy
7	female	13 months	Beagle	Healthy
8	female	1 year	Beagle	Healthy

PBMC culture and treatment

PBMC fraction was separated by density gradient centrifugation using Lymphoprep reagent and SepMate-15 tubes (STEMCELL, #07851 and #85415) according to the manufacturer's protocol. The cells were centrifuged, resuspended in RPMI-1640 medium with 10% FBS and Pen-Strep antibiotic mix, and seeded at the initial concentration of 10^5 cells/well, 50µl/well, on a 96-well culture plate. The cells from each animal were grown either exposed to no additional factors, ConcanavalinA (ConA; 5µg/ml; ThermoFisher, #00-4978-03), or ConA and one of the three antibodies at 10µg/ml: PD1-1.1, PD1-2.1, isotype control (Biolegend, #401509). The culture

lasted 72h, after which the plate was centrifuged. The conditioned medium from each well was harvested and transferred to a prepared ELISA plate in the next step.

PBMC IFN-γ ELISA

The Quantikine Canine IFN-γ Immunoassay kit (R&D Systems. #CAIF00), which applies a sandwich enzyme immunoassay technique, was utilized in this study to quantify IFN-γ. The kit was used in accordance with the manufacturer's protocol. Briefly, the provided canine IFN-γ standard was reconstituted and two-fold serial dilution were prepared, starting at a concentration of 4000 pg/mL, to generate the standard curve. Following the preparation of capture antibody-coated wells, the prepared samples and standard solutions were added. This step was followed by an incubation period. Subsequently, the wells were washed to remove unbound substances. Then, a detector antibody against canine IFN-γ conjugated with biotin was added. The detector antibody binds to the antigen already captured by the first antibody, effectively sandwiching the antigen between the capture antibody and the detector antibody. After another incubation period, another wash followed. The wells were then treated with Streptavidin-HRP (Horseradish Peroxidase), which binds to the Biotin Conjugate, forming an antigen-antibody-enzyme complex. This was followed by an additional incubation and washing phase. The Substrate Solution, which reacts with HRP, resulting in a color change, was then added. The reaction was stopped by adding the Stop Solution, which halts color development. The solution's absorbance was measured at a wavelength of 450 nm with a correction at 570 nm. In four cases the reader noted signal overflow, resulting in missing data; those PBMC samples were rejected, leaving PBMC samples from 5 dogs in the analysis. The mean and standard deviation were calculated for experimental duplicates. The averaged signal was normalized through dividing by the signal from isotype control wells of each respective PBMC sample.

PBMC ELISA statistical analysis

Significant differences in signal between treatment groups were sought. The t-test was initially considered for this analysis. However, the t-test assumes that the data is normally distributed, and upon evaluation using the Shapiro-Wilk test, it was determined that the data was not normally distributed for some of the treatment groups. Consequently, the Kruskal-Wallis non-parametric test, which does not assume normal distribution, was performed using GraphPad Prism software and revealed a significant difference to be present among some of the treatment groups. To

identify these groups, a post-hoc analysis was conducted using Dunn's multiple comparisons test, which adjusts the p-values for multiple comparisons, reducing the risk of false positive results. Despite these efforts to employ robust statistical methods, the analysis yielded some unexpected results. Differences were observed between certain treatment groups, but some comparisons that appeared to show biologically meaningful differences did not reach statistical significance. Working with small sample sizes presents challenges, as it diminishes the statistical power of conventional analyses. Consequently, it is essential to interpret results by considering both statistical significance and biological relevance.

Supplementary comments

PBMC ELISA

Unexpectedly, some PBMC samples stimulated with Concanavalin A (ConA) alone exhibited higher signal than those treated with ConA and the isotype control, which puts the assay mechanism in question. Curiously, a similar issue was observed in the paper by Coy, where addition of isotype control to activated PBMCs significantly lowered the signal. Unlike us, Coy et al. detected statistically significant upregulation of IFN-γ secretion upon treatment with both their mAbs. Nemoto et al. similarly to us observed inconsistent results between dogs. They have seen more positive trends than us, though the visualization of results did not allow for the assessment of statistical significance. The current lack of standardization together with the previously listed weak points of a PBMC IFN-γ assay appear to limit its usefulness.

In more detail, there were several issues encountered with our PBMC PD-1 blockade assay that merit discussion. First, we observed substantial heterogeneity in the immune responses of PBMCs to the test antibodies PD1-1.1 and PD1-2.1. While one dog's PBMCs showed a strong response consistent with our previous ELISA results, this outcome was an outlier, with most dogs showing no discernible response. Due to the varying medical histories and treatments of these dogs (information not disclosed to us), we could not control for potential factors that might influence the immune state of the PBMCs. These factors could potentially affect activation or exhaustion of lymphocytes, proportion of immune cell populations, expression of PD-1 and PD-L1, inflammation or immune suppression. Although the ConA control validated that T-cells in the PBMC samples were capable of activation and IFN-gamma production, it could not account for

all the possible complex immune states of the PBMC population. Thus, we acknowledge that the results may not be generalizable.

Second, we noted an unexpected result where the signal from PBMCs treated with ConA alone was higher than that of ConA combined with any of the antibodies. This finding contradicts the expected outcome based on the assay mechanism. Under theoretical expectations, PBMCs activated with ConA alone should produce a signal similar to those treated with both ConA and the isotype control, while PBMCs treated with PD-1 blocking antibodies should display an elevated signal due to the removal of immune inhibition. It is conceivable that PBMCs treated with blocking antibodies might not display an enhanced signal compared to ConA alone if the antibodies are not effective blockers or if there is an insufficient level of PD-L1 expression in the PBMCs to permit PD-1 blocking. However, that fails to explain why the signal for all antibodies was even lower than for ConA treatment. This discrepancy may be attributed to several factors:

- Non-specific binding of antibodies: the antibodies may have non-specifically bound to PBMC components, potentially altering the immune response.
- Fc receptor interactions: the tested antibodies could interact with Fc receptors on immune cells, modulating immune responses. As murine antibodies are used in the assay along canine cells, cross-species reactivity further complicates these interactions.
- Mixed PBMC population: while the assay and its interpretation focus on cytotoxic T-cells, a dominating constituent of PBMC fraction, the presence of various other immune cell types, including T cells, B cells, NK cells, monocytes, and dendritic cells, may impact the overall immune response.

In summary, while the PBMC-based IFN-gamma assay aims to simulate a more realistic immune environment than simpler *in-vitro* assays, its interpretability appears severely limited. A more controlled approach could involve isolating cytotoxic T-cells followed by adding recombinant PD-L1 for standardized inhibition, or co-isolating T-cells and PD-L1 expressing cells. The cells could be isolated by magnetic or flow cytometry-based cell sorting. Investigation of Fc receptor interactions between the murine antibodies and canine cells used in the assay, as well as the impact of various cell populations in the PBMC fraction could be labor intensive, but informative for future studies.

Finally, one limitation specific to our assay was found late. We employed one isotype control antibody of IgG2a isotype, while PD1-1.1 and PD1-2.1 were of IgG2a and IgG2b subtypes, respectively. While the a/b difference may not be relevant in most molecular assays, it could have an impact on an immune cell-based assay. In this context, the key distinction between IgG2a and IgG2b lies in their ability to engage the immune system. Specifically, IgG2a is generally considered more pro-inflammatory due to its enhanced ability to interact with Fc receptors and activate immune cells, whereas IgG2b is less potent in this regard. Although the exact interaction between murine isotypes and canine Fc receptors is uncertain, we hypothesize that they could bind differentially. In such a case results for PD1-1.1 would remain comparable to the isotype control, but those for PD1-2.1 less so. Consequently, the experiment should be repeated with improved controls.
